# HVEM Gene Polymorphisms Are Associated with Sporadic Breast Cancer in Chinese Women

**DOI:** 10.1371/journal.pone.0071040

**Published:** 2013-08-16

**Authors:** Dalin Li, Zhenkun Fu, Shuang Chen, Weiguang Yuan, Yanhong Liu, Liqun Li, Da Pang, Dianjun Li

**Affiliations:** 1 Department of Breast Surgery, The Third Affiliated Hospital of Harbin Medical University, Harbin, China; 2 Department of Immunology & Heilongjiang Provincial Key Laboratory for Infection and Immunity, Harbin Medical University, Harbin, China; 3 Institute of Cancer Prevention and Treatment, Harbin Medical University, Harbin, China; 4 Department of Laboratory Medicine, The Second Hospital of Harbin Medical University, Harbin, China; University of North Carolina School of Medicine, United States of America

## Abstract

As a costimulatory molecule, Herpesvirus entry mediator (HVEM) can bind with several costimulatory members, thus HVEM plays different roles in T cell immunity. HVEM and its ligands have been involved in the pathogenesis of various autoimmune, inflammatory diseases and tumors. In the current study, we conducted a case-control study comparing polymorphisms of HVEM and breast cancer. Subjects included 575 females with breast cancer and 604 age-matched healthy controls. Six HVEM SNPs (rs2281852, rs1886730, rs2234163, rs11573979, rs2234165, and rs2234167) were genotyped by PCR-RFLP. The results showed significant differences in genotypes and alleles between rs1886730 and rs2234167 (*P*<0.05). One haplotype (CTGCGG) that was associated with breast cancer was found via haplotype analysis. Our research also indicated an association between polymorphisms of HVEM and clinicopathologic features, including lymph node metastasis, estrogen receptor, progesterone receptor and P53. Our results primarily indicate that polymorphisms of the HVEM gene were associated with the risk of sporadic breast cancer in northeast Chinese females.

## Introduction

Costimulatory molecules are an important second signal and are required to promote T cell activation, survival and differentiation, and to advance cytokine mediated clonal expansion. Coinhibitory molecules can transduce negative signals. This contributes to the attenuation of initial T cell activation and modulates the process of T cell differentiation by limiting T cell proliferation and survival. Coinhibitory and costimulatory molecules can be divided into two major super families according to their structures: the immunoglobulin superfamily (IgSF) and the tumor necrosis factor/tumor necrosis factor receptor superfamily (TNF/TNFRSF) [1]. Herpesvirus- entry mediator (HVEM, also called “herpesvirus entry mediator A and tumor necrosis factor receptor superfamily member 14), a 283 amino acid single-pass type I membrane protein that belongs to TNFRSF, is expressed prominently in hematopoietic cells and lymphoid tissues [2], [3]. HVEM protein can bind types 1 and 2 herpesvirus glycoprotein D (gD) allowing entry into the related cells. X-ray crystallography has shown that HVEM can bind to a flexible hairpin at the amino terminus of gD [4], [5] and to other costimulatory members known as LIGHT (TNFSF14 or CD258), BTLA (CD272) and CD160 [6]–[8]. Early research has shown that the interaction between HVEM and LIGHT can induce costimulatory signals leading to T cell activation and regulation of IFN-γ production [9], [10]. In contrast, HVEM-BTLA engagement produces proinflammatory signals leading to NF-κB activation, and can participate in the inhibition of T cell activation [11]. The combination of HVEM and its ligands provides a highly regulated bidirectional mechanism that modulates cell survival, activation or attenuation of the immune response [7], [8], [12].

Single nucleotide polymorphisms (SNPs) represent a natural genetic variability with a great number present in the human genome. SNPs usually occur more frequently in non-coding regions than in the coding regions, where natural selection is acting and fixating the allele of the SNP that constitutes the most favorable genetic adaptation [13]. SNP variations in human DNA sequences can affect human disease and response to pathogens, chemicals, drugs, vaccines, and other agents. SNPs are also thought to be important in personalized medicine [14]. The HVEM gene is located on chromosome 1p36, which contains other TNFRSF members, such as 4-1BB and OX40. Until now, research on the polymorphisms of HVEM has been limited to HSV infection [15], and the relationship between polymorphisms of HVEM and breast cancer has not yet been investigated.

In recent years, there has been an increase in female breast cancer in developing countries. Cell-mediated immunity plays an important role in combating breast cancer, and co-stimulatory molecules play significant roles in the initial stage of the cellular immune response. In the current study, we investigated the association between the polymorphisms of costimulatory molecule–HVEM and female sporadic breast cancer in Northeast China.

## Materials and Methods

### Subjects

A total of 575 female subjects with breast cancer (age 49.5±10.17) and 604 healthy controls (age 46.2±9.6) were included in the study. All of the sporadic breast cancer cases, were recruited from the Third Affiliated Hospital of Harbin Medical University, China and were diagnosed by histopathological confirmation. Clinical features of breast cases, including pathological type, tumor size, lymph node metastasis, human epidermal growth factor receptor 2 (C-erbB2), estrogen receptor (ER), progesterone receptor (PR) and protein 53 (P53) statuses are shown in Table 1. All healthy female controls were recruited randomly from a community in the same district. None of the controls had a history of personal malignancy or autoimmune disorder and were frequency-matched to cases by age. The ethical board from the Third Affiliated Hospital of Harbin Medical University approved the study before beginning any research and all of the volunteers gave written confirmed consent.

**Table 1:** Clinicopathologic information of breast cancer patients.

Clinicopathologic information	Case No.(%)
Tumor type	
IDC	492(85.57)
MC	5(0.87)
Intraductal carcinoma	40(6.96)
Mucinous adenocarcinoma	14(2.43)
others	24(4.17)
Tumor Size(cm)	
Less than 2	193(33.56)
2 to 5	260(45.22)
More than 5	28(4.87)
Unknown	94(16.35)
LN involvement	
Positive	242(42.09)
Negative	322(56.00)
Unknown	11(1.91)
**ER**	
Positive	287(49.91)
Negative	207(36.00)
Unknown	81(14.09)
**PR**	
Positive	354(61.57)
Negative	138(24.00)
Unknown	83(14.43)
**P53**	
Positive	148(25.74)
Negative	331(57.57)
Unknown	96(16.69)
**CerbB-2**	
Positive	186(32.35)
Negative	305(53.04)
Unknown	84(14.61)

IDC infiltrative ductal carcinoma, MC medullary carcinoma, LN lymph node, TZ tumor size, ER estrogen receptor, PR progesterone receptor.

### SNP selection

SNPs of HVEM gene were selected for study using the base of the HapMap database. Six tag SNPs, including rs2281852, rs1886730, rs2234163, rs11573979, rs2234165 and rs2234167, with pair-wise r^2^>0.8 for each SNP pair were chosen from HapMap data using Haploview 4.0 software (**Figure S1**). The minor genotype frequency of six SNPs was more than 1% in the Chinese Han (CHB) population. All of the SNP information regarding the HVEM gene was acquired from the dbSNP database (http://www.ncbi.nlm.nih.gov/projects/SNP/) and Hapmap (http://hapmap.ncbi.nlm.nih.gov/).

### DNA extraction and genotyping

Genomic DNA was extracted from whole blood using a Universal Genomic DNA Extraction Kit Ver. 3.0 (TaKaRa, Japan). All genotyping of the 6 SNPs was performed by polymerase chain reaction restriction fragment length polymorphism (PCR-RFLP) assay. The polymorphic region was amplified by PCR using a T-Gradient Thermoblock PCR System (Biometra, Germany). A 25 ul reaction solution containing 0.3 ug genomic DNA, 2.5 ul 10× PCR buffer (Mg^2+^ plus), 0.2 ul dNTPs mixture (2.5 uM), 2.5 U TaqDNA polymerase (TaKaRa, Japan) and 0.2 ul of each primer (10 uM) (Invitrogen, China) was used. The primers, restriction enzymes, length of PCR products and digested fragments for HVEM PCR-RFLP genotyping are shown in Table 2. Annealing temperatures were 58.2°C (rs2281852), 56°C (rs1886730), 58°C (rs2234163), 56°C (rs11573979), 55°C (rs2234165) and 59°C (rs2234167). The accuracy of genotyping results was confirmed using direct sequencing in random samples.

**Table 2:** Primers and PCR programs for HVEM PCR-RFLP genotyping.

SNP	primer	restriction enzyme	PCR products length	Length of digested fragments
rs2281852	F: 5′-CCTACCTGCCTCTGCCATTG-3′	DdeI	186bp	A:101+85bp
	R: 5′-AGGGCTTCGTTGATGGGAG-3′			C: 186bp
rs1886730	F: 5′-TCCCACAGATCTCTTCCC-3′	BsmFI	678bp	C: 263+226+97+50+42bp
	R: 5′-CTGGGAACTGGAACTCTGC-3′			T: 489+97+50+42bp
rs2234163	F: 5′-TTGGCCTGTGGATGCTGTC-3′	HpyCH4III	245bp	A: 129+116bp
	R: 5′-CGCTTACCTCCCTTCTGCAC-3′			G: 245bp
rs11573979	F: 5′-CCTACCTGCCTCTGCCATTG-3′	NlaIII	292bp	T: 220+47+25bp
	R: 5′-AAACGAGGTGCCCAGAGGTAC-3′			C: 267+25bp
rs2234165	F: 5′-CAGACCAAGTAAGTGAACCC-3′	BfaI	221bp	A: 129+92bp
	R: 5′-TCTGATGAGGCTTTGTCTGGG-3′			G: 221bp
rs2234167	F: 5′-ACCGCTGTGAGACCATTG-3′	FokI	270bp	A: 172+98bp
	R: 5′-GGGTTCTTTCCTGAGCTAC-3′			G: 270bp

### Statistical analysis

Genotype frequencies for the 6 SNPs were tested for Hardy-Weinberg equilibrium (HWE) among the breast cancer cases and healthy controls. Haploview 4.1 software was used to tag all common haplotypes, and their frequencies, in both breast cancer cases and controls. Associations between SNPs and breast cancer risk were estimated by odds ratio (OR) and 95% confidence interval (CI). Disease characteristics were compared among patients using the chi-square test. Homozygotes for the major allele were used as the reference group, and the heterozygotes and minor allele homozygotes were compared with the reference group. Comparisons of the distributions of the allele, genotype and haplotype frequencies were performed using the chi-square test and statistical significance was set at P<0.05. Statistical analyses were performed using SPSS 16.0 software.

## Results

### Genotypes and Alleles

The frequencies of genotypes and alleles of the 6 SNPs in the HVEM gene for both breast cancer patients and healthy controls are shown in Tables 3 and 4. All genotypes of the 6 SNPs were in accordance with Hardy–Weinberg equilibrium in the breast cancer case and control groups (P>0.05). There was a statistically significant difference in the distribution of the genotype rs1886730 when comparing breast cancer case and control groups (additive P = 0.001719, dominant P = 0.0003795, recessive P = 0.0008484). There was a lower frequency of heterozygotes of rs2234167 in the breast cancer case group than in the control group (P = 0.000432) (**Table S1**). The T allele (P = 0.0000142) in rs1886730 and the A allele in rs2234167 (P = 0.000622) were also lower in breast cancer cases than in controls (Table 4). The P value for alleles of rs1886730 and rs2234167 were corrected for multiple testing, using 10,000 permutations, by the Haploview program and the differences were significant (P = 0.0001 and 0.0031). However, there were no statistically significant differences in genotypes or alleles in rs2281852, rs2234163, rs2234165 and rs11573979 (P>0.05).

**Table 3:** Genotype frequencies of HVEM polymorphisms and their associations with breast cancer risk.

SNP	Minor,(a)	Major,(A)	Cases			Controls			P value for model of inheritance		
			‘AA’	‘Aa’	‘aa’	‘AA’	‘Aa’	‘aa’	Additive	Dominant	Recessive
rs2281852	A	C	181	303	81	199	315	90	0.8278	0.5896	0.6595
rs1886730	T	C	208	251	105	163	276	161	**0.0001719**	**0.0003795**	**0.0008484**

Rs2281852: cases n = 575, missing n = 0; controls n = 604, missing n = 0.

Rs1886730: cases n = 564, missing n = 11; controls n = 600, missing n = 4.

Minor allele ‘a’ and the major ‘A’ are shown in the table. ‘AA’, ‘Aa’, ‘aa’ represent a given variant for each SNP genotyped. Numbers in the columns marked “cases” and “controls” are the numbers of each class of genotype. Significant values (p<0.05) are in bold.

**Table 4:** Allele frequencies of HVEM polymorphisms and their associations with breast cancer risk.

SNPs of HVEM	Alleles	NO. (%)		OR (95% CI)	P value
		Cases(n = 575)	Controls(n = 604)		
Rs2281852	C	665(57.83%)	713(59.02%)	Reference	
	A	485(42.17%)	495(40.98%)	1.051(0.892–1.238)	0.555
Rs1886730	C	667(59.13%)	602(50.17%)	Reference	
	T	461(40.87%)	598(49.83%)	**0.696(0.591**–**0.820)**	**0.0000142** *
Rs2234163	G	1114(97.72%)	1184(98.34%)	Reference	
	A	26(2.28%)	20(1.66%)	1.382(0.767–2.489)	0.280
Rs11573979	C	1140(99.82%)	1197(99.58%)	Reference	
	T	2(0.18%)	5(0.42%)	0.420(0.081–2.169)	0.285
Rs2234165	G	1125(98.00%)	1184(98.50%)	Reference	
	A	23(2.00%)	18(1.50%)	1.345(0.722–2.505)	0.349
Rs2234167	G	1109(96.60%)	1129(93.46%)	Reference	
	A	39(3.40%)	79(6.54%)	**0.509(0.344–0.754)**	**0.000622** *

* P<0.01 after correcting the P value for multiple testing by Haploview program using 10,000 permutations.

Rs2281852: cases n = 575, missing n = 0; controls n = 604, missing n = 0.

Rs1886730: cases n = 564, missing n = 11; controls n = 600, missing n = 4.

Rs2234163: cases n = 570, missing n = 5; controls n = 602, missing n = 2.

Rs11573979: cases n = 571, missing n = 4; controls n = 601, missing n = 3.

Rs2234165: cases n = 574, missing n = 1; controls n = 601, missing n = 3.

Rs2234167: cases n = 574, missing n = 1; controls n = 603, missing n = 1.

### Haplotype analysis

The associations between haplotypes of the HVEM gene and breast cancer were confirmed using Haploview software; the frequencies of haplotypes were greater than 1% (Table 5). The most frequent haplotype that appeared in breast cancer cases and controls was CTGCGG (rs2281852-rs1886730-rs2234163-rs11573979-rs2234165-rs2234167) (31.0%) though it had a significantly lower frequency in breast cancer cases (P = 0.0078). The frequencies of haplotypes ACGCGG (rs2281852-rs1886730-rs2234163-rs11573979-rs2234165-rs2234167) and CCGCGG (rs2281852-rs1886730-rs2234163-rs11573979-rs2234165-rs2234167) were significantly higher in breast cancer cases compared with controls (P = 0.0126, P = 0.01, respectively). But after correcting the P value for multiple testing by Haploview program using 10,000 permutations, we found that only CTGCGG (rs2281852-rs1886730-rs2234163-rs11573979-rs2234165-rs2234167) had statistical difference in all haplotypes (P = 0.0482).No statistically significant difference was found after analyzing other haplotypes (P>0.05).

**Table 5:** Haplotypes of HVEM gene.

HVEM Haplotypes						Frequency	Cases (n = 575)	Controls (n = 604)	P value	Permutation P value*
S1	S2	S3	S4	S5	S6					
C	T	G	C	G	G	0.310	0.284	0.335	0.0078	**0.0482**
A	C	G	C	G	G	0.275	0.299	0.253	0.0126	0.0900
C	C	G	C	G	G	0.220	0.243	0.199	0.0100	0.0638
A	T	G	C	G	G	0.105	0.096	0.113	0.1693	0.7530
C	T	G	C	G	A	0.015	0.010	0.019	0.0869	0.5198
A	C	G	C	G	A	0.012	0.010	0.015	0.3050	0.9619
C	C	G	C	G	A	0.012	0.008	0.016	0.0977	0.5643

S1 = rs2281852, S2 = rs1886730, S3 = rs2234163, S4 = rs11573979, S5 = rs2234165, S6 = rs2234167.

* correcting the P value for multiple testing by Haploview program using 10,000 permutations.

### Clinical features

In the current study, the association between HVEM polymorphisms and a series of clinicopathologic features in breast cancer case information were identified. These included lymph node metastasis, tumor size (maximum diameter), ER, PR, P53 and CerbB-2 statuses. There was no statistical difference between HVEM polymorphisms and tumor size. In rs2281852, we found that the AA genotype (P = 0.00465) and the A allele (P = 0.016) were significantly less frequent in cases of lymph node metastasis; similar results were also found in PR positive cases (P = 0.044, P = 0.030, respectively). In rs1886730, we found that the CT (P = 0.006) genotype was appeared more frequently in lymph node metastasis positive cases. The CT (P = 0.016) and TT (P = 0.006) genotypes and T allele (P = 0.002) were also more frequent in CerbB-2 positive cases. In ER positive breast cancer patients, we observed that the AG genotype (P = 0.006) and A allele (P = 0.007) were more frequent in rs2234167. In P53 positive cases, we found that the AG genotype (P = 0.016) and A allele (P = 0.017) were occurred more often in rs2234163.

The association analysis between HVEM haplotypes and breast cancer clinicopathologic features found that haplotype ACGCGG (rs2281852-rs1886730-rs2234163-rs11573979-rs2234165-rs2234167) was occurred more frequently in lymph node metastasis, PR and CerbB-2 negative cases (P = 0.0187, P = 0.043 and P = 0.0383, respectively). Haplotype CTGCGA (rs2281852-rs1886730-rs2234163-rs11573979-rs2234165-rs2234167) was more frequent in ER positive cases (P = 0.0291) and haplotype CTGCGG (rs2281852-rs1886730-rs2234163-rs11573979-rs2234165-rs2234167) had a higher incidence in CerbB-2 positive cases (P = 0.0060). However, no significant associations were observed between haplotypes and P53.

## Discussion

Researching genetic polymorphisms is a new approach that is used to investigate the etiology of complex diseases. As an inherited disorder, breast cancer has been associated with immunological factors. The IgSF is an important group of co-stimulatory molecules that participate in T cell immune response and the development of breast cancer. HVEM is a member of the IgSF. It is activated through the binding of BTLA, LIGHT and CD160 [6], [16]. BTLA binds the N-terminal cysteine-rich domain of HVEM [17] and the BTLA-HVEM interaction can suppress the function of Treg cells [18]. Our previous research showed that BTLA polymorphisms were associated with the risk of breast cancer [19]. In the current case-control study, 6 SNPs were selected to cover the HVEM gene region using Hapmap data and the relationship between HVEM gene polymorphisms and sporadic breast cancer in Northeast Chinese women was examined.

We found a lower frequency of the CT heterozygote, the TT homozygote and the T allele, located in the intron, of rs1886730, in breast cancer patients. This indicates that these variations of rs1886730 may have protective effects against breast cancer. Introns play vital roles in transcription and RNA stability [20], thus mutations in introns may lead to the disruption of the splice site, causing the splicing enhancement, silence or alteration of the mRNA secondary structure [21]. Based on these facts, it is possible that rs1886730 may have effects on the transformation of splicing in HVEM.

rs2234167 was located in the exon of the HVEM gene, where mutations can lead to the amino acid substitution from isoleucine (A) to valine (G). In rs2234167, we found that the AG heterozygote and the A allele played protective roles in sporadic breast cancer. The mutation of rs2234167 may influence the binding affinity between HVEM and BTLA/LIGHT/CD160. rs2234167 in the exon of the HVEM gene may affect the function and expression of HVEM during the appearance or development of breast cancer.

Steroid hormone receptors are valuable for the prediction and prognosis of breast cancer and are regarded as predictive markers for endocrine therapy, especially for ER and PR [22]–[24]. Analysis comparing clinical features and HVEM gene polymorphisms found that the AA genotype and the A allele of rs2281852 occurred less frequently in PR positive cases, while the AG genotype and the A allele were more frequent in rs2234167. The CT and TT genotypes and the T allele of rs1886730 were all more frequent in CerbB-2 positive cases and the AG genotype and A allele were more frequent in P53 positive cases in rs2234163. The expression of CerbB-2 and the mutation of P53 can lead to tumor metastatsis, insensitivity to endocrine treatment and poor prognosis [25], [26]. The oncogene CerbB-2 and suppressor gene P53 play important roles in the treatment and prognosis of breast cancer. Therefore, polymorphisms of rs2234163 and rs1886730 may be important in forecasting the prognosis of breast cancer and the effectiveness of hormonal treatment.

In order to further identify the relationship between HVEM gene polymorphisms and sporadic breast cancer, haplotypes of the HVEM gene were analyzed using Haploview software. According to our results, the block CTGCGG (rs2281852-rs1886730-rs2234163-rs11573979-rs2234165-rs2234167) had the highest frequency (31.0%) among all haplotypes. The frequency of haplotype CTGCGG (rs2281852-rs1886730-rs2234163-rs11573979-rs2234165-rs2234167) was lower in breast cancer cases, so this haplotype may be a protective factor in breast cancer among Chinese females. Associations were also found between haplotypes and clinical features including lymph node metastasis, PR, ER and CerbB-2 statuses in our research, and these haplotypes also had higher frequencies among all haplotypes. So these haplotypes may be important in the prediction of breast cancer and may be the valuable prognostic factors for survival.

## Conclusion

Our current work indicates that HVEM gene polymorphisms may affect the susceptibility to sporadic breast cancer risk in women of the northeast of China. To our knowledge, this is the first evidence for the involvement of the human HVEM gene in breast cancer. The analysis of breast cancer clinical features in our study revealed an association between HVEM gene polymorphisms and some prognostic factors in breast cancer, such as the statuses of ER, PR, CerbB-2 and P53.

## Supporting Information

Figure S1 **SNP selection.**
(TIF)

Table S1 **Genotype frequencies of HVEM polymorphisms and their associations with breast cancer risk.**
(DOC)
