# Supplementary figures and images for: HVEM Gene Polymorphisms Are Associated with Sporadic Breast Cancer in Chinese Women
Source: PLoS One. 2013 Aug 16;8(8):e71040. doi: 10.1371/journal.pone.0071040 (PMC3745383; doi:10.1371/journal.pone.0071040)

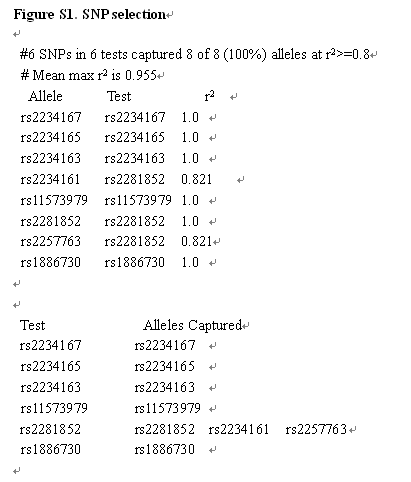

Supplement: Figure S1 — SNP selection. (TIF) [file pone.0071040.s001.tif]
