# Supplementary material for: HVEM Gene Polymorphisms Are Associated with Sporadic Breast Cancer in Chinese Women
Source: PLoS One. 2013 Aug 16;8(8):e71040. doi: 10.1371/journal.pone.0071040 (PMC3745383; doi:10.1371/journal.pone.0071040)
Supplement: Table S1 — Genotype frequencies of HVEM polymorphisms and their associations with breast cancer risk. (DOC) [file pone.0071040.s002.doc]

**Table S1. Genotype frequencies of HVEM polymorphisms and their associations with breast cancer risk**

(There were only one homozygote in these four SNPs loci of HVEM)

| SNPs of HVEM | Genotypes | NO. (%) | | OR (95% CI) | P value |
| --- | --- | --- | --- | --- | --- |
|  |  | Cases(n=575) | Controls(n=604) |  |  |
| Rs2234163  Exon | GG | 544(95.44%) | 582(96.68%) | Reference |  |
|  | AG | 26(4.56%) | 20(3.32%) | 1.391 (0.676-2.520) | 0.275 |
| Rs11573979  Intron | CC | 569(99.65%) | 596(99.17%) | Reference |  |
|  | CT | 2(0.35%) | 5(0.83%) | 0.419(0.081-2.168) | 0.285 |
| Rs2234165  Intron | GG | 551(95.99%) | 583(97.00%) | Reference |  |
|  | AG | 23(4.01%) | 18(3.00%) | 1.352 (0.722-2.533) | 0.345 |
| Rs2234167  Exon | GG | 535(93.21%) | 525(86.92%) | Reference |  |
|  | AG | 39(6.79%) | 78(13.08%) | **0.491(0.328-0.734)** | **0.000432** |

Rs2234163 cases n=570, missing n=5; controls n=602, missing n=2

Rs11573979 cases n=571, missing n=4; controls n=601, missing n=3

Rs2234165 cases n=574, missing n=1; controls n=601, missing n=3

Rs2234167 cases n=574, missing n=1; controls n=603, missing n=1
